# Backward logistic regression analysis of the determinants of the hand function among patients with leprosy: A cross-sectional study

**DOI:** 10.51866/oa.405

**Published:** 2024-03-30

**Authors:** Rizky Kusuma Wardhani, Melinda Harini, Fitri Anestherita, Febrina Nur'Alfiah Ramadhani

**Affiliations:** 1 MD, Department of Physical Medicine and Rehabilitation, Dr. Cipto Mangunkusumo Hospital National General Hospital, Faculty of Medicine, Universitas Indonesia, Jl. Pangeran, Diponegoro No.71, Kenari, Kec., Senen, Central Jakarta, Jakarta, Indonesia. Email: Rizkykusumawardhani@yahoo.co.id; 2 MD, Department of Physical Medicine and Rehabilitation, Dr. Cipto Mangunkusumo Hospital National General Hospital, Faculty of Medicine, Universitas Indonesia, Jl. Pangeran Diponegoro No.71, Kenari, Kec. Senen, Central Jakarta, Jakarta, Indonesia.; 3 MD, Department of Physical Medicine and Rehabilitation, Dr. Cipto Mangunkusumo Hospital National, General Hospital, Faculty of Medicine, Universitas Indonesia, Jl. Pangeran Diponegoro No.71, Kenari, Kec. Senen, Central Jakarta 10430, Jakarta, Indonesia.; 4 MD, Faculty of Medicine Universitas, Indonesia, Jl. Salemba Raya No.6, Kenari, Kec. Senen, Central Jakarta, Jakarta, Indonesia.

**Keywords:** Leprosy, Hand deformities, Disability

## Abstract

**Introduction:**

The hands are the most common site of disability in leprosy. Hand dysfunction could result in difficulty performing activities of daily living. Therefore, hand function should be regularly assessed to ensure that any decrease in hand function could be diagnosed earlier.

**Methods:**

This study included 110 patients with leprosy from Likupang and Lembata, Indonesia. Hand function was assessed using the modified Jebsen test to measure hand function respective of the dominance. The grip and pinch strength were used as objective measures of clinical arm function. The World Health Organization (WHO) hand disability grade were used to determine the degree of impairment. Other factors such as age, sex and the type of leprosy were also considered. All factors were analysed using backward logistic regression.

**Results:**

Among the 110 participants, a decrease in the dominant (48.2%) and non-dominant (50.9%) hand functions were found. Pinch strength (OR: 3.39; 95% CI: 1.13–10.19) and age (OR: 4.91; 95% CI: 1.72–14.03) were significantly associated with hand function irrespective of the dominance. Conversely, the WHO hand disability grade (OR: 2.97; 95% CI: 1.10–8.04) and type of leprosy (OR: 0.34; 95% CI: 0.12–0.97) were significantly associated with only function of the dominant hand.

**Conclusion:**

There is a significant association of age and pinch strength with hand function regardless of the hand dominance. In contrast, the WHO hand disability grade and type of leprosy are significantly associated with the function of the dominant hand only.

## Introduction

In 2021, South East Asia accounted for 66.7% (n=93,485) of new leprosy cases globally, with 10,976 new cases in Indonesia. This statistic showed a slight decrease from the previous year, which was 11,173 new cases.^1^ Leprosy or Hansen’s disease is a chronic infectious disease caused by *Mycobacterium leprae* and *Mycobacterium lepromatosis* The disease mainly affects mucocutaneous tissues and peripheral nerves, causing loss of sensation in the skin and, possibly, the development of deformities and disability during its progression.^4^ Hands are the most common site of disability in leprosy, with about 44.48%–49% of patients with leprosy experiencing hand deformities, such as claw hands or contractures.^5,6^ Hand dysfunction may lead to dependency in activities of daily living, consequently increasing the social and economic burden.^7,8^

As hands are the most common site of disability among patients with leprosy, regular evaluation is needed to assess its function. There are many instruments for objectively assessing hand function, and one of the most used instruments is the Jebsen test of hand function (JTHF).^9^ The modified Jebsen test (MJT) is a shorter version of JTHF used to assess the gross functional dexterity of the upper extremity in moderately impaired individuals. The original JTHF consists of seven subsets, while the modified version comprises only three subsets that simulate everyday activity, making it rather easier to administer in daily practice.^10,11^

Few studies have assessed the hand function of patients with leprosy in daily activities and its related factors. Previous studies have mostly focused on nerve damage in patients’ hands rather than functional dexterity.^12,13^ This study is aimed to evaluate hand function of patients with leprosy, mainly its functional dexterity, and its associated factors. By knowing the hand function of patients and its associated factors, more comprehensive rehabilitation treatment to retain function can be given to patients.

## Methods

This cross-sectional study included 110 patients with leprosy. Data were obtained during a collaborative study programme called ‘Katamataku’ involving an ophthalmologist, an otolaryngologist, a dermatologist and physiatrists from the University of Indonesia, Jakarta, Indonesia. The programme was held at St Damian Hospital Lembata, North Nusa Tenggara, Indonesia, and Puskesmas Likupang, North Minahasa, North Sulawesi, Indonesia, in August 2022. Participants needed to meet the following inclusion criteria: 1) retrospective diagnosis of leprosy, 2) complete identity, 3) completion of all required tests and 4) absence of a history of hand amputation.

There were 156 participants during the event, but only 110 patients met the inclusion criteria. Demographic and clinical information was collected through direct history-taking with patients. The WHO hand disability grade was assessed by dermatologists and physiatrists. The pinch strength and grip strength were also measured in both hands by physiatrists as objective measurements for clinical arm function.^12,14^ The hand functions were measured by physiatrists using the MJT. The examiners were not blinded in this study.

### Measurements

The MJT consists of three tasks conducted in order of performance: flipping five cards upside down, stacking four cones and spooning five kidney beans into a bowl. In this study, the objects were placed on a horizontal board (85x32 cm and a 9-cm-high ridge at the back of the board) in a standardised manner at table height. Markings were made on the board to indicate the position of the cards, cones and beans, with the centre of the objects 15 cm apart. The MJT was performed in both the dominant hand and non-dominant hand, with the dominant hand tested first.^12^ The time taken to perform all tasks for each hand was recorded using a stopwatch. Since there were no normative data found regarding the MJT, the median of the data was calculated using SPSS Statistics for Windows version 24 (IBM Corp. Released 2016. IBM SPSS Statistics for Windows, Version 24.0. Armonk, NY: IBM Corp). and a cut-off time was set –21.19 s for the dominant hand and 24.94 s for the nondominant hand.

The WHO hand disability grade was assessed as follows: grade 0: no anaesthesia, no visible deformity or damage in the hands; grade 1: anaesthesia present, no visible deformity or damage in the hands; or grade 2: visible hand deformity or damage present.

The grip strength and pinch strength were used as objective measures of upper extremity integrity. The grip strength was measured using a Jamar hand dynamometer, with the base of the dynamometer resting on the heel of the palm and the handle on the middle of the four fingers. Participants were then asked to squeeze the dynamometer with maximum effort. Conversely, the pinch strength was measured via the key pinch method, wherein the thumb pad presses on the lateral aspect of the middle phalanx on the index finger, using a pinch metre. These measurements were then compared with the normative data.^14,15^

### Data management and analysis

The collected data were checked and processed in Microsoft Excel. The data were then exported to SPSS for statistical analysis. For analysis, the data were converted into nominal form by dividing the data of each variable into two categories. For the MJT score, pinch strength and grip strength, the data were categorised based on the measurements on the dominant hand and non-dominant hand. Furthermore, as the dependent variable, the MJT score was then divided into above and below the cut-off. The pinch strength and grip strength were also divided into above and below the cut-off based on the normal value. For the WHO hand disability grade, the data were divided into no deformity (grade 0) and anaesthesia or deformity present (grades 1 and 2). For age, the data were divided into 20–60 years and >60 years.

The data were checked for normal distribution using the Kolmogorov–Smirnov test: The data for all variables were found to be non-normally distributed (sig<0.000). The MJT scores of the dominant hand and non-dominant hand were then separately analysed using the chi-square independence test to determine the significance of each independent variable (WHO hand disability grade, pinch strength and grip strength) and confounding variable (age, sex and type of leprosy). After bivariate analysis, multivariate analysis of all variables for each dominant hand or non-dominant hand was performed using backward logistic regression. The variables that were found to be insignificant were then eliminated one by one, and the elimination process stopped when all variables became significant.

## Results

Descriptive analysis was conducted first before further analysis using backward logistic regression. The demographic and clinical characteristics of the participants, including age, sex, educational level, type of leprosy and WHO hand disability grade, are shown in Table 1. The mean age of the 110 participants was 49 years.

**Table 1:** Characteristics of the patients with leprosy from Likupang and Lembata.

**Characteristics**		**n**	**%**
**Age**	20–30 years	17	15.5
	31–40 years	15	13.6
	41–50 years	31	28.2
	51–60 years	20	18.2
	>60 years	27	24.5
**Sex**	Male	74	67.3
	Female	36	32.7
**Residence**	Likupang	28	25.5
	Lembata	82	74.5
**Educational level**	No education	9	8.2
	Primary school	48	43.6
	Middle school	21	19.1
	High school	27	24.5
	Bachelor’s degree and above	5	4.6
**Type of leprosy**	Paucibacillary	24	21.8
	Multibacillary	86	78.2
**WHO hand disability grade**	Grade 0	80	72.7
	Grade 1	19	17.3
	Grade 2	11	10.0
**Dominant hand**	Right	78	70.9
	Left	32	29.1

Table 2 presents the descriptive analysis of the variables. For the statistical analysis, age and the WHO hand disability grade were divided into two categories only. For the grip strength, 70 (63.6%) participants in the dominant hand group and 82 (74.5%) participants in the non-dominant hand group scored below the normal value. For the pinch strength, 23 (20.9%) participants in the dominant hand group and 42 (38.2%) participants in the non-dominant hand group scored below the normal value. For the MJT score, 53 (48.2%) participants in the dominant hand group and 56 (50.9%) participants in the non-dominant hand group finished the test above the cut-off time.

**Table 2:** Descriptive analysis of the hand function and its determinants among the patients with leprosy.

**Variables**		**n**	**%**
**Age**	20-60 years	83	75.5
	>60 years	27	24.5
**Sex**	Male	74	67.3
	Female	36	32.7
**Type of leprosy**	Paucibacillary	24	21.8
	Multibacillary	86	78.2
**WHO hand disability grade**	Grade 0	80	72.7
	Grades 1 and 2	30	27.3
**Hand grip strength – dominant hand**	Below the normal value (Abnormal)	70	63.6
	Above the normal value (Normal)	40	36.4
**Hand grip strength – non-dominant hand**	Below the normal value (Abnormal)	82	74.5
	Above the normal value (Normal)	28	25.5
**Hand pinch strength – dominant hand**	Below the normal value (Abnormal)	23	20.9
	Above the normal value (Normal)	87	79.1
**Hand pinch strength – non-dominant hand**	Below the normal value (Abnormal)	42	38.2
	Above the normal value (Normal)	68	61.8
**Modified Jebsen test score – dominant hand**	Above the cut-off (Abnormal)	53	48.2
	Below the cut-off (Normal)	57	51.8
**Modified Jebsen test score – non-dominant hand**	Above the cut-off (Abnormal)	56	50.9
	Below the cut-off (Normal)	54	49.1

All variables were then analysed using backward logistic regression to eliminate those that were not significantly related. The backward logistic regression analysis of the function of the dominant hand is presented in Table 3. Among the variables, the pinch strength, WHO hand disability grade, age and type of leprosy were all found to be significantly related to the function of the dominant hand. Age was found to be the most significant factor, followed by the pinch strength, WHO hand disability grade and type of leprosy. An increase in age was associated with a greater risk of decrease in the function of the dominant hand (OR: 4.91; 95% CI: 1.72–14.03). A decrease in the pinch strength of the dominant hand was associated with a greater risk of a decrease in the function of the dominant hand (OR: 3.39; 95% CI: 1.126–10.194). An increase in the WHO hand disability grade was related to an increase in the risk of a reduced function of the dominant hand (OR: 2.97; 95% CI: 1.10–8.04). Lastly, multibacillary (MB) leprosy was related to a higher risk of a reduced function of the dominant hand (OR: 0.34; 95% CI: 0.12-0.97).

**Table 3:** Backward logistic regression analysis of the modified Jebsen test score of the dominant hand.

		**B**	**SE**	Sig.	**Exp (B)**	**95% CI for Exp (B)**	
						Lower	Upper
Step 1	**Pinch strength (dominant hand)**	1.342	0.582	0.021	3.82	1.22	11.98
	**Grip strength (dominant hand)**	-0.678	0.478	0.156	0.51	0.20	1.30
	**WHO hand disability grade**	1.204	0.522	0.021	3.34	1.20	9.28
	**Sex**	-0.128	0.486	0.793	0.88	0.34	2.28
	**Age**	1.487	0.545	0.006	4.43	1.52	12.89
	**Type of leprosy**	-0.865	0.552	0.117	0.42	0.14	1.24
Step 2	**Pinch strength (dominant hand)**	1.319	0.573	0.021	3.74	1.22	11.49
	**Grip strength (dominant hand)**	-0.685	0.477	0.152	0.50	0.20	1.29
	**WHO hand disability grade**	1.200	0.522	0.021	3.32	1.19	9.23
		1.478	0.543	0.006	4.39	1.51	12.71
	**Type of leprosy**	-0.894	0.541	0.098	0.41	0.14	1.18
Step 3	**Pinch strength (dominant hand)**	1.220	0.562	**0.030**	3.39	1.13	10.19
	**WHO hand disability grade**	1.088	0.508	**0.032**	2.97	1.10	8.04
		**B**	**SE**	Sig.	**Exp (B)**	**95% CI for Exp (B)**	
						Lower	Upper
Step 1	**Pinch strength (dominant hand)**	1.342	0.582	0.021	3.82	1.22	11.98
		1.591	0.536	**0.003**	4.91	1.72	14.03
	**Type of leprosy**	-1.067	0.530	**0.044**	0.34	0.12	0.97

Contrary to the function of the dominant hand, only two variables were found to be significantly related to the function of the non-dominant hand: pinch strength (OR: 2.39; 95% CI: 1.04-5.49) and age (OR: 4.75; 95% CI: 1.71-13.23) (Table 4).

**Table 4:** Backward logistic regression analysis of the modified Jebsen test score of the nondominant hand.

		**B**	**SE**	Sig.	**Exp (B)**	**95% CI for Exp (B)**	
						Lower	Upper
Step 1	**Pinch strength (non-dominant hand)**	0.733	0.465	0.115	2.08	0.84	5.18
	**Grip strength (non-dominant hand)**	0.531	0.510	0.298	1.70	0.63	4.62
	**WHO hand disability grade**	0.250	0.518	0.629	1.28	0.47	3.54
	**Sex**	-0.124	0.453	0.784	0.88	0.36	2.15
	**Age**	1.710	0.557	0.002	5.53	1.86	16.47
	**Type of leprosy**	-0.262	0.512	0.608	0.77	0.28	2.10
Step 2	**Pinch strength (non-dominant hand)**	0.730	0.465	0.116	2.08	0.84	5.16
	**Grip strength (non-dominant hand)**	0.522	0.508	0.304	1.69	0.62	4.56
	**WHO hand disability grade**	0.242	0.517	0.640	1.27	0.46	3.51
		**B**	**SE**	Sig.	**Exp (B)**	**95% CI for Exp (B)**	
						Lower	Upper
Step 1	**Pinch strength (non-dominant hand)**	0.730	0.465	0.115	2.08	0.84	5.18
	**Age**	1.702	0.555	0.002	5.48	1.85	16.29
	**Type of leprosy**	-0.292	0.500	0.559	0.75	0.28	1.99
Step 3	**Pinch strength (non-dominant hand)**	0.813	0.430	0.059	2.25	0.97	5.24
	**Grip strength (non-dominant hand)**	0.512	0.506	0.312	1.67	0.62	4.50
	**Age**	1.729	0.554	0.002	5.63	1.90	16.68
	**Type of leprosy**	-0.253	0.492	0.607	0.78	0.30	2.04
Step 4	**Pinch strength (non-dominant hand)**	0.809	0.430	0.060	2.25	0.97	5.21
	**Grip strength (non-dominant hand)**	0.494	0.505	0.328	1.64	0.61	4.41
	**Age**	1.692	0.549	0.002	5.43	1.85	15.93
Step 5	**Pinch strength (non-dominant hand)**	0.871	0.424	**0.040**	2.39	1.04	5.49
	**Age**	1.559	0.522	**0.003**	4.75	1.71	13.23

## Discussion

This study revealed that among the 110 participants, 48.2% and 50.9% had a decreased fUnction of their dominant hand and nondominant hand, respectively. In the dominant hand, age, the pinch strength, the WHO hand disability grade and the type of leprosy were found to be significantly related to the hand function. In the non-dominant hand, only two factors were found to be significantly related to the hand function: age and pinch strength. Sex and the grip strength were found to be insignificant factors irrespective of the hand dominance.

In this study, the hand function was measured using the MJT. In general, this test evaluates the gross functional dexterity of the hand respective of its dominance. Dexterity is defined as the ability to perform skilful fine motor functions of the hand, which are mostly done by the dominant hand.^16^ The hand function and dexterity largely determine the quality of performance of activities of daily living, work-related activities and recreational activities.^17^ In leprosy, the fine motor ability is disrupted due to damage in the ulnar nerve, median nerve or both. Impairment of the ulnar nerve combined with the median nerve on the dominant side could affect the pinch strength and performance of basic daily activities at a highly significant level.^18^

Age was found to be the strongest factor influencing the hand function of the patients with leprosy irrespective of the dominance in the present study. Multiple studies have found that the frequency of neuropathy increases significantly among older adults.^19-21^ This might be because age and the duration of the disease are directly related. Because leprosy is a chronic disease, patients can experience more disability due to disease progression over time.^22^ Moreover, the hand function can also decrease due to the nature of ageing, especially in older adults.^17^ Therefore, old age in combination with the disability caused by leprosy might cause further hand dysfunction.

Herein, the pinch strength was also found to be related to the hand function regardless of the dominance. In contrast, the grip strength was not related to the hand function regardless of the dominance. These findings are in line with the report by Rajkumar et al. that only the pinch strength was found to be significantly related to the performance of basic activities of daily living (BADLs).^22^ Another study also found that the grip strength and pinch strength were significant in seven out of eight BADLs in the dominant hand, whereas the pinch strength was found to be related only in two out of eight BADLs in the non-dominant hand.^18^

In this study, approximately 72.7% of the participants had a WHO hand disability grade of 0. We believe that this is because 75% of our participants belonged to the younger age group (20-60 years), with a mean age of 49 years. This finding agrees with a previous report that age is one of the factors related to the WHO hand disability grade.^23^ Multiple other studies have also noted that age is associated with hand disability.^24-26^

Supporting the present findings, other studies have also shown that the hand disability grade is significantly related to activity limitation.^16,27^ Although the WHO hand disability grade is found to be significantly associated with the hand function, the use of this parameter has some limitations. This grading system is not sensitive to change after treatment; hence, it is not suitable for evaluating the effectiveness of treatment.^27^

An interesting finding in this study is that 40% (n=32) of the participants in the grade 0 disability group showed a decreased function of the dominant hand (P=0.005) based on their MJT score. Further studies are needed to better understand this finding. However, based on this finding, we recommend a regular hand function assessment in patients with leprosy even with a disability grade of 0.

The type of leprosy was also associated with the function of the dominant hand of the participants in this study. The participants with MB leprosy showed a significantly reduced function of their dominant hand. To date, limited studies have directly evaluated the association between the hand function and type of leprosy among patients. In the meta-analysis by de Paula et al., patients with MB leprosy were found to be four times more likely to experience disability. This is probably attributed to the higher bacilli loads in patients who have MB leprosy due to the absence of humoral immune response.^28^

In this study, 67.3% of the participants were men. No significant association was noted between sex and the hand function irrespective of the dominance. In contrast, de Paula et al. suggested that men are almost twice more likely to experience physical disability in leprosy than women.^28^ This high risk for disability in men is thought to be due to social behaviour and both reluctance and difficulty in accessing healthcare facilities.^29^

Further studies that would assess the hand function of patients with leprosy or even perhaps develop a hand function assessment tool that could be easily administered and interpreted by any healthcare professionals are needed. As early diagnosis is a critical factor in preventing disability, earlier recognition of a decreased hand function would mean earlier treatment and prevention of further disability.

The study has some limitations that should be considered. The first one is the lack of data about the duration of leprosy, history of leprosy reaction and history of treatment that might affect the disability outcome in patients and, hence, the function of their hands. The second one is the limited number of patients, limiting the generalisation of the findings.

## Conclusion

Based on the results and discussion above, there is a significant association of age and the pinch strength with the hand function among patients with leprosy in Likupang and Lembata regardless of their hand dominance. Conversely, the WHO hand disability grade and type of leprosy are significantly associated with the function of the dominant hand only. There is also a possibility of a decreased hand function even with a WHO hand disability grade of 0; hence, we recommend regular assessment of the hand function even in patients with no anaesthesia or deformity.

## Data sharing statement

All raw data are encrypted with passwords and are not publicly available.

**How does this paper make a difference in general practice?**

- The study identified several factors associated with the hand function of patients with leprosy. With these findings, clinicians can arrange an appropriate treatment plan to prevent further disability.
- Disability in leprosy is formerly only measured using the WHO hand disability grade, and it is uncommon to check for the hand function if there are no lesions or anaesthesia. However, this study found that even in patients with a WHO hand disability grade of 0, there is a possibility of a decreased hand function. Hence, regular assessment of the hand function is needed to detect and prevent further disability.
